# Factors associated with secondhand tobacco smoke in the home: an exploratory cross-sectional study among women in Aleta Wondo, Ethiopia

**DOI:** 10.1186/s12889-016-3588-6

**Published:** 2016-08-31

**Authors:** Anne Berit Petersen, Lisa M. Thompson, Gezahegn Bekele Dadi, Alemu Tolcha, Janine K. Cataldo

**Affiliations:** 1 Center for Tobacco Control Research and Education, University of California, San Francisco, 530 Parnassus Avenue, Suite 366, San Francisco, CA 94143 USA; 2 Department of Family Health Care Nursing & Global Health Sciences Program, University of California, San Francisco, 2 Koret Way, Nursing, Rm 405J, UCSF Box 0606, San Francisco, CA 94143 USA; 3 School of Nursing and Midwifery, College of Medicine and Health Sciences, Hawassa University, PO Box 1560, Hawassa, Ethiopia; 4 School of Public & Environmental Health, College of Medicine and Health Sciences, Hawassa University, PO Box 1560, Hawassa, Ethiopia; 5 Department of Physiological Nursing & Center for Tobacco Control Research and Education, 2 Koret Way, N611Q, San Francisco, CA 94143 USA; 6 Loma Linda University School of Nursing, West Hall, 11262 Campus St, Loma Linda, CA 92354 USA

**Keywords:** Tobacco, Secondhand smoke, Smoke-free homes, Women, Ethiopia, Urbanicity, *khat* use, Household decision-making, Multidimensional Poverty Index

## Abstract

**Background:**

In Ethiopia, female smoking rates are currently low (1 %). However, because of male smoking rates (overall 7.7 % and up to 27 % depending on region), women and children’s risk of second hand smoke (SHS) exposure is a pressing concern. In order to develop effective public health interventions that prevent the uptake and exposure to smoking, thereby averting the projected increase in tobacco-induced disease, an understanding of Ethiopian women’s practices regarding tobacco is needed. The purpose of this study was to explore Ethiopian women’s tobacco use and prevalence of SHS exposure, and to identify covariates associated with SHS exposure.

**Methods:**

We conducted an exploratory cross-sectional study in Southern Ethiopia between August and October 2014, and systematically sampled households in Aleta Wondo town and surrounding districts. Trained interviewers verbally administered surveys to women 18–55 years of age. Descriptive statistics and multiple logistic regression analyses were performed.

**Results:**

None of the 353 participants reported current tobacco use and less than 1 % reported ever use, however, 11 % reported ever use of the stimulant leaf *khat*. Twenty-seven women (7.6 %) reported living with a tobacco user, however, twice that number (14.4 %) overall, and 22 % of urban participants reported that smoking occurred daily in their home. When controlling for other factors, living with a tobacco user (OR = 9.91, 95 % CI [3.32, 29.59]), allowing smoking in the home (OR = 5.67, 95 % CI [2.51, 12.79]), place of residence (OR = 2.74, 95 % CI [1.11, 6.74)]), and exposure to point-of-sale advertising within the last 30 days (OR = 2.87, 95 % CI [1.26, 6.54]) contributed significantly to a model predicting the likelihood of reporting daily occurrence of smoking/SHS in the home.

**Conclusions:**

While few women reported having ever used tobacco, one in seven women in this study reported that smoking/SHS occurred daily in their homes. Therefore SHS exposure is a potential health concern for women and children in this rural community. Findings from this study provide baseline data for monitoring tobacco control policies in Ethiopia, particularly in relation to the promotion of smoke-free homes, and could be used to inform prevention program development.

## Background

Among the world’s developing regions, sub-Saharan Africa has recently experienced the highest rate of increase in tobacco use and it is projected that by 2030 this region will emerge as the epicenter of the tobacco epidemic [1]. Factors contributing to this projection include the rising rate of adolescent smoking in sub-Saharan African countries (overall 9 % of boys and 3 % of girls, and as high as 30.7 % in Madagascar and 11.2 % in Nambia, respectively), rapid population growth and increased consumer purchasing power [1, 2]. Additionally, Africa is currently one of the prime market targets of the tobacco industry and the industry’s influence has been posited as the greatest obstacle to implementation of proactive tobacco control measures in this region [2–5].

Ethiopia is one country in the region that is currently experiencing considerable flux in relation to tobacco. In January 2014, the Ethiopian parliament ratified the World Health Organization Framework Convention on Tobacco Control (WHO FCTC), and during the same year moved to privatize shares of the National Tobacco Enterprise, which up until November of 2014 had been a legal tobacco monopoly [6, 7]. The recent bidding war by the large tobacco companies for a major share of the Ethiopian tobacco monopoly is indicative the phenomenal growth being projected for this market [8].

An increase in tobacco use across sub-Saharan Africa, and in Ethiopia specificially, will disproportionately impact women and children. Tobacco use, including SHS exposure, not only increases women’s risk of non-communicable diseases (NCDs) (e.g., cardiovascular disease, chronic obstructive pulmonary disease, and cancer), it also includes associated risks for unborn children (e.g. premature delivery, stillbirth) and infants (e.g. low birth weight, sudden-infant death syndrome, acute lower respiratory infections) [2]. Additionally, based on research conducted in other LMICs, women are more likely to be impacted by the burden of caring for family members with tobacco-related diseases, and income spent on tobacco products instead of necessities such as food, healthcare and education, adversely affects women and children’s health and well-being [2, 9–11]. Therefore, increasing our understanding of women’s behaviors related to tobacco use and factors associated with SHS exposure in the home is critical to the development of proactive and comprehensive tobacco control policy and interventions.

In Ethiopia, prevalence data on smoking and SHS exposure among women are extremely limited, particularly among those living in rural settings (versus major metropolitan areas) —where more than 80 % of the population currently lives [12]. Yet, the increased prevalence of substance use, particularly among youth, of tobacco and the stimulant-containing *khat* leaf (*catha edulis*; a plant-based alkaloid stimulant that is native to Ethiopia and typically chewed) have become urgent public health problems [12–14]. A number of studies conducted in Ethiopia have demonstrated higher cigarette consumption with *khat* use [13, 14]. Smoking is purported to intensify the stimulant effect of *khat*, and help in the alleviation of the negative psychological effect that occurs when the stimulant wears off [15]. The use of *khat* is legal in most of Ethiopia, and tends to be a highly social behavior [12, 16]. For women, *khat* use is more socially acceptable than tobacco, representing a potential gateway for tobacco use, as has been described in other both high and low-income settings [17–20]. In Ethiopia, dual use with *khat* has been presented as a possible explanation for the higher levels of tobacco use in rural settings, where *khat* use also tends to be higher [15, 21, 22].

While the national smoking prevalence is currently low among women (1 %), due to the current and projected prevalence of smoking among men, women’s risk of SHS exposure is a pressing concern [2]. According to the 2011 Ethiopian Demographic Health Survey (EDHS), the overall cigarette smoking prevalence among men is 7.7 %, however it varies significantly by age (11–13 % for 40–49 years) and region of the country (up to 27 %) [12]. In an Eastern Ethiopian cross-sectional, sub-regional study conducted in a rural town, smoking prevalence among men was as high as 38.6 and 33 % of the respondents reported daily occurrence of indoor household smoking (i.e., SHS) [15]. Finally, unlike patterns in other sub-Saharan countries, there is evidence to suggest that adult smoking in Ethiopia may be higher in rural versus urban populations for both men and women (21.6 % vs.10.8 % and 1.1 % vs. 0.7 % respectively) [23]. The projected increases in tobacco use across the sub-Saharan African region, the increasing prevalence of smoking by males in Ethiopia, and the documented expansion of the tobacco industry create significant risks for increased SHS exposure among women and children in Ethiopia [1, 3, 24]. In preparation for the burgeoning tobacco epidemic and in order to develop effective public health interventions to address these risks, research to understand Ethiopian women’s potential risk of SHS exposure is needed now. The purpose of this study was to explore Ethiopian women’s tobacco use and prevalence of SHS exposure, and to identify covariates associated with SHS exposure.

## Methods

### Sample

From August to October 2014, a community based cross-sectional exploratory study was conducted in Aleta Wondo town and its outlying *kebeles* (smallest administrative units) located within the Southern Nations, Nationalities, and Peoples’ Region (SNNPR) in Ethiopia. The region was selected in consultation with collaborating research partners, including Hawassa University and Common River (501C non-governmental agency working in the region), and was based on jurisdiction and accessibility. The Aleto Wondo *woreda* (district) has a total of 9 *kebeles*, however, because of accessibility, the study sample was drawn from four *kebeles*, two in the rural town and two in the surrounding rural area. Utilizing census reports provided by *kebele* leaders, the sample was drawn systematically, with 25 % being drawn from each of the four *kebeles*.

In the *Demographic Health Surveys*, definitions of urban and rural are country specific [25]. Using the 2005 EDHS classification, the entire region would be classified as rural; however, the data obtained at the local administrative level distinguishes *kebeles* based on location (i.e. either within the rural town or in the outlying rural areas) [26]. In contrast to the urban *kebeles*, the rural *kebeles* are notably less densely populated and lack most forms of infrastructure (e.g., lack of motorable roads, limited access to electricticy and absence of commercial shops, businesses, banks, retail kiosks). In order to allow for exploration of differences between a gradient of “urbanicity” (i.e. the degree to which a geographical unit is urban), in this study we use “urban” to denote a place of residence in a *kebele* located within the rural town, and “rural” for a place of residence in one of the outlying *kebeles* [27]. The inclusion criteria for this study were: (a) being an Ethiopian woman 18 to 55 years of age, (b) living in Ethiopia continuously for the past 5 years, (c) having children or grandchildren 12 years old or younger currently living in the household, (d) having the ability to communicate verbally in either the Amharic and/or the Sidama dialects, and (e) being a primary cook in the household. Participants were excluded if they were deemed mentally incompetent and unable to complete the survey, as determined by the interviewer.

### Measures

A structured questionnaire, developed by Bloch et al. to study knowledge, attitudes, and behaviors related to tobacco use and SHS exposure among pregnant women in low and middle-income countries (LMICs), was adapted for use in this study [28]. Additional items were drawn from the *Global Adult Tobacco Survey* [29]. The study questionnaire included questions that addressed women’s use of tobacco, knowledge of tobacco-related health hazards, perception of social acceptability of tobacco use by women, perceived benefits of tobacco use, whether smoking was allowed indoors in the home, frequency of smoking in the home (proxy for SHS exposure), children’s exposure to combustible tobacco, non-personal use of tobacco, household involvement in the tobacco industry, and exposure to pro- and anti-tobacco messages. In addition, the survey included questions to evaluate women and children’s potential concurrent exposure to household air pollution from household cooking fires. Demographic characteristics were included and incidence and intensity measures of acute poverty were calculated using the Global Multidimensional Poverty Index (MPI), which takes into account the multiple direct sources of deprivation at the household level [30]. The MPI indicators are distributed in three weighted dimensions of poverty: health, education, and standard of living, and the measure defines a household as “multidimensionally poor” if they are deprived in 33 % or more of the weighted indicators [30]. Finally, a 3-item version of the Household Decision-Making scale (HDM) was included to measure women’s perceived involvement in household decision-making [31]. The HDM evaluates an individual’s participation in decisions concerning household purchases (i.e., major and daily neccesities) and visitation of friends and relatives. The participant indicates whether the type of decision is made by themselves (self) or jointly with a partner, and has been used to predict married women’s involvement in a range of health behaviors in LMICs [32–34].

#### Adaptation and translation of questionnaires

The WHO guidelines for translation and adaptation of instruments and recommendations from the International Agency for Research on Cancer were used to guide the development of a culturally appropriate instrument [35, 36]. First, a template of the survey was developed in English and reviewed for clarity and appropriateness by local health professionals with direct knowledge of the target population. Language experts, with expertise in English and the two primary dialects spoken in the study area (Sidama and Amharic), conducted forward translations of the survey from English to the respective dialects. After the translations were complete, expert bilingual panels were convened, which included translators, health experts, local educators, representatives from the collaborating NGO, and one of the co-primary investigators (Co-PI). The goal of these panels was to resolve inadequate expressions and concepts of translation [35]. Once this input was integrated, language experts who had no prior knowledge of the instrument provided a back-translation of the survey from the respective dialects to English. Discrepancies were discussed with members of the expert panels in an iterative process until final versions were agreed upon.

#### Pre-testing

To ensure equivalence of question comprehension and meaning, the survey questions were verbally administered in the Sidama dialect in two focus groups with women local community (*n* = 17) [35]. Participants were asked to explain what the questions were asking, repeat the question in their own words, and describe what came to mind when they heard key terms or phrases. Additionally, they were asked to indicate if there were any words to which they did not understand the meaning and any expressions that they found unacceptable or offensive. The Amharic version was pre-tested for clarity in individual interviews (*n* = 5).

#### Selection and training of interviewers

Two local residents, both native Sidama and Amharic speakers, were trained to conduct the interviews. Written protocols for administering the survey were developed and pre-tested in collaboration with the interviewers. During the training process, minor modifications to the procedures were made based on field experiences.

#### Recruitment procedures and sampling technique

A systematic sampling technique was employed in this study [37]. Interviewers were assigned to a *kebele* and instructed to start in the geographical center of the *kebele*. Randomized numbers (1 to 10) were used in the selection of the first house. After the first house, every third house was selected radiating out in four different directions in the *kebele*. Guides who were familiar with each *kebele* were hired to accompany the interviewers and to assist with navigation to the boundaries of each *kebele*.

The prospective participant was approached at her home and invited to participate using a script. If no one met the eligibility requirements, the interviewers went to the next immediate house. If more than one woman in a household met the inclusion criteria, the primary cook was selected. As per the request of Common River, our collaborating NGO, in lieu of an incentive (e.g., money or gift item) participants were given paper invitations to a community educational event which included a health education presentation on the health consequences of both tobacco and cooking fire smoke, recommended prevention strategies, an overview of the study findings, and a “feast” meal.

#### Data analysis

Descriptive statistics were generated for all variables. Group comparisons were made by place of residence (urban vs. rural) and by potential for SHS exposure (daily vs. non-daily occurrence of smoking/SHS in the home) (outcome variable). Groups were compared using Independent *t*-tests and Chi-square or Fisher’s Exact Tests. Bivariate analysis was conducted among factors that were theorized to have potential associations with reports of daily occurrence of smoking/SHS in the home. Nine independent variables were significant at the <0.10 level (age, house-hold decision making, place of residence, ethnicity, tobacco user in household, household member involved in growing, selling or manufacturing of tobacco products, smoking allowed in home (proxy for lack of household smoking ban), exposure to point-of-sale advertising, and ever tried *khat*) and were selected for inclusion in model development. Using a criterion of <0.05 *p*-value, multiple logistic regression was performed to assess the impact of these variables and potential interaction terms on the likelihood that participants would report daily occurrence of smoking/SHS in their homes. Both unadjusted and adjusted odds ratios (AOR) are reported. The data was analyzed using the SPSS Version 22 statistical software and differences at the 0.05 alpha level are reported [38].

## Results

A total of 708 households were contacted; 137 were not at home, and among the 372 eligible participants, 16 declined to participate, and three were not fluent in either Sidama or Amharic. This resulted in 353 completed surveys; 78.2 % of which were conducted in the Sidama dialect and 21.8 % in Amharic. The cooperation rate among eligible participants was 95.2 % [39]. The mean (SD) reported age was 29.4 years (6.9), the majority of the sample was married (94.9 %), identified their tribal association as Sidama (71.5 %), were Protestant (79.2 %), and more than 50 % of the sample reported having completed 5 years or less of formal education (Table 1). Among the represented households, 13.6 % were classified as “multidimensionally poor” (*n* = 48), and the average intensity of deprivation among the individuals in these households (*n* = 271) was 0.74 (i.e., deprived in 74 % of the indicators). When participants were compared by place residence, those residing in the urban *kebeles* had significantly higher levels of education, tended to be more ethnically and religiously diverse, reported higher levels of shared decision-making, were more likely to rent the dwelling they lived in, and overall had significantly improved MPI indicators, as compared to those residing in the rural *kebeles* (Table 1).

**Table 1:** Sample characteristics and differences by place of residence (*N* = 353)

	Total	Rural	Urban	*p*
		(*n* = 179)	(*n* = 174)	
Maternal age (years), mean, ± SD (*n* = 347)	29.4 ± 6.9	29.0 ± 6.5	29.7 ± 7.3	.074
Education (years), mean, ± SD	5.7 ± 3.8	4.3 ± 3.1	7.1 ± 4.0	< .001
Total # persons in household, mean, ± SD	5.8 ± 2.0	6.1 ± 2.1	5.4 ± 1.8	.037
Crowding (# persons/# rooms), mean, ± SD	2.6 ± 1.6	2.8 ± 1.8	2.4 ± 1.3	.022
	*n* (%)	*n* (%)	*n* (%)	
Marital status				.051
Married	334 (94.9)	174 (97.8)	160 (92.0)	
Other	18 (5.1)	4 (2.2)	14 (8.0)	
Currently pregnant	31 (8.8)	16 (8.9)	15 (8.6)	ns
Ethnicity (tribal association)				< .001
Sidama	251 (71.5)	167 (93.8)	84 (48.6)	
Amhara	58 (16.5)	6 (3.4)	52 (30.1)	
Gurage	20 (5.7)	2 (1.1)	18 (10.4)	
Oromo	14 (4.0)	3 (1.7)	11 (6.4)	
Other	8 (2.3)	1 (0.0)	8 (4.6)	
Religious affiliation, type				< .001
Protestant	278 (79.2)	174 (97.8)	104 (60.1)	
Orthodox Christian	61 (17.4)	3 (1.7)	58 (33.5)	
Muslim	10 (2.8)	0 (0)	10 (5.8)	
Catholic	2 (0.6)	1 (0.6)	1 (0.6)	
Multidimensional Poverty Index (MPI)				
“Multidimensionally poor” households^a^	48 (13.6)	37 (20.7)	11 (6.3)	<.001
Individual MPI Indicators:^b^				
No one in household completed 5 years schooling	38 (11.3)	29 (16.6)	9 (5.6)	.002
School-age child not enrolled in grade 1 to 8	31 (8.8)	22 (12.4)	9 (5.2)	.018
Child death (< 5 years of age)	107 (30.3)	56 (31.3)	51 (29.3)	ns
No electricity	71 (20.1)	57 (31.8)	14 (8.0)	<.001
Limited access to clean drinking water^c^	85 (24.1)	50 (27.9)	35 (20.1)	.055
No toilet or improved latrine	64 (18.1)	16 (8.9)	48 (27.6)	<.001
Dirt, sand or dung flooring in dwelling	150 (42.5)	117 (65.4)	33 (19.0)	<.001
Cook with biomass fuel (wood, charcoal, or dung)	352 (99.7)	178 (99.4)	174 (100)	ns
Assets^d^	244 (69.1)	153 (85.5)	91 (52.3)	<.001
Type of house				<.001
Rented, leased or borrowed	91 (25.8)	4 (2.2)	87 (50.0)	
Owned	262 (74.2)	175 (97.8)	87 (50.0)	
Household decision-making (HDM)				
Composite HDM score^e^, mean ± SD	1.7 ± 1.2	1.5 ± 1.2	2.0 ± 1.1	<.001

^a^Number and percent (%) households deprived in ≥33.3 % of weighted MPI indicators

^b^Number and percent (%) deprived within each individual indicator

^c^Household does not have access to clean drinking water or clean water is > 30 min walk from home

^d^Household does not own more than one of: radio, TV, telephone, bike, or motorbike, and do not own a car or tractor

^e^Composite score of three HDM questions. Score for each question = 1 if reported “self” or “jointly” (Possible score 0 to 3 with higher scores associated with greater participation in decision-making)

### Tobacco and *kha*t use

Less than 1 % of the sample reported ever smoking cigarettes or trying smokeless products and none reported current use of any tobacco products (Table 2). When queried on intention to try either cigarettes or smokeless tobacco within the next year, more than 97 % responded “definitely not” and less than 3 % reported “definitely” or any ambivalence (i.e., “maybe” or “don’t know”) about trying either type of product. No significant differences were observed between patterns of tobacco use in rural and urban *kebeles*; however, a significant difference was observed in relation to reporting ever use of *khat* between urban (20.1 %, *n* = 35) versus rural participants (2.2 %, *n* = 4, *p* < .001).

**Table 2:** Rural and urban differences in tobacco and khat use, secondhand smoke exposure, and exposure to point-of-sale advertising (*N* = 353)

	Total		Rural	Urban	*p*
	*n*	%	(*n* = 179)	(*n* = 174)	
Cigarette use					
Never smoker	350	(99.2)	177 (98.9)	173 (99.4)	ns
Ever tried but not smoking now	3	(0.8)	2 (1.1)	1 (0.6)	ns
Smokeless tobacco product use					
Ever tried but not currently using (chewing tobacco)	2	(0.3)	1 (0.6)	1 (0.6)	ns
*Khat* use					
Ever chewed	39	(11.0)	4 (2.2)	35 (20.1)	<.001
Chewed in last 30 days (% yes)	12	(3.4)	1 (0.6)	11 (6.3)	ns
Secondhand smoke exposure in home					
Live with one or more tobacco users^a^	27	(7.6)	9 (5.0)	18 (10.3)	.060
Smoking of tobacco products permitted indoors^b^	51	(14.6)	13 (7.3)	38 (21.9)	<.001
Smoking occurs daily inside house^c^	50	(14.4)	11 (6.1)	39 (23.1)	<.001
Young children (≤5 years) frequently/always exposed to tobacco smoke indoors^d^	11	(5.1)	6 (5.2)	5 (5.0)	ns
Member of household currently involved in growing, manufacturing, or selling tobacco products (% yes)	21	(5.9)	10 (5.6)	11 (6.3)	ns
Exposure to point-of-sale advertising, in last 30 days (% yes)	191	54.1	73 40.8	89 51.1	.055

^a^Participants were asked, *“How many people living in your household use tobacco products?”*

^b^Participants were asked, *“I want to ask you about smoking inside you house. Please answer from the following options. Inside your house smoking is 1) allowed, 2) not allowed, but there are exceptions, 3) never allowed.”* (Response 1 or 2 = permitted)

^c^Participants were asked, *“How often does someone smoke inside your house? (daily, weekly, monthly, less than monthly, or never)”*

^d^Results include only respondents with children 5 years or younger (*n* = 215; Rural = 116, Urban = 99)

### Secondhand smoke exposure

Among the total sample, 7.6 % of participants (*n* = 27) reported living in a household where at least one member was a current tobacco user, 14.6 % (*n* = 51) reported that smoking was allowed in their homes, and 14.4 % of participants (*n* = 50) reported that smoking occurred “daily” in their homes (Table 2). When participants were asked about how often their young children (≤ 5 years) were in close proximity inside the home to people using combustible tobacco, 5.1 % (*n* = 20) reported that their young children were exposed to SHS “frequently” or “always” (Table 2). Urban residents were more likely to report that smoking was allowed in the home than rural residents (21.8 % vs. 7.3 %, *p* < .001), and nearly four times more likely than rural residents (*p* < .001) to report daily occurrence of smoking/SHS in their homes.

When a simultaneous multiple logistic regression was performed to assess the impact of the nine significant variables identified in the bivariate analysis on the likelihood that participants would report daily occurrence of smoking/SHS in the home, the full model was statistically significant, *χ*^2^ (9, *N* = 338) = 83.10, *p* < .001, -2*LL* = 200, and explained 38.4 % (Nagelkerke pseudo-R square) of the variance in the daily occurrence of smoking/SHS in the home (Table 3). Only four of the independent variables made a unique statistically significant contribution to the model. The strongest predictor of reporting daily occurrence of smoking/SHS in the home was having a member of the household who is currently using tobacco products, with an AOR of 9.91, 95 % CI [3.32, 29.59], followed by smoking allowed in the home [AOR 5.67, 95 % CI (2.51, 12.79)], exposure to point-of-sale advertising within the last 30 days [AOR 2.87, 95 % CI (1.26, 6.54)], and residing in an urban setting [AOR 2.74, 95 % CI (1.11, 6.74)].

**Table 3:** Bivariate and multivariate logistic regression predicting likelihood of women in Aleta Wondo, Ethiopia reporting daily occurrence of smoking/SHS in the home [*n* = 338]

	Unadjusted OR [95 % CI]^a^	*p* value	Adjusted OR [95 % CI]^b^	*p* value
Maternal age^c^	1.04 [0.99, 1.08]	.09	1.01 [0.96, 1.07]	.65
Household Decision-making^d^	1.26 [0.96, 1.65]	.09	0.91 [0.64, 1.28]	.57
Place of residence (urbanicity)^e^	4.58 [2.26, 9.29]	<.001	2.74 [1.11, 6.74]	.03
Ethnicity [tribal association] ^f^	3.56 [1.92, 6.60]	<.001	1.44 [0.59, 3.49]	.42
Member of household is a current user of tobacco products	12.28 [6.13, 24.58]	<.001	9.91 [3.32, 29.59]	<.001
Member of household involved in growing, manufacturing or selling of tobacco products	12.28 [5.27, 28.61]	.10	2.67 [0.81, 8.79]	.11
Smoking allowed in home (No home smoking ban)^g^	2.57 [0.95–6.98]	<.001	5.67 [2.51, 12.79]	<.001
Exposure to point-of-sale tobacco advertising, within last 30 days^h^	2.02 [1.10, 3.72]	.03	2.87 [1.26, 6.54]	.01
Ever use *khat* ^h^	2.04 [0.90, 4.61]	.09	1.14 [0.41, 3.15]	.80

^a^Significance *p* < .10

^b^Significance *p* < .05

^c^Continuous variable (years). Six ‘Don’t know’ responses were excluded

^d^Continuous variable (composite HDM score)

^e^Reference category: Residing in a rural district (versus urban)

^f^Reference category: Sidama (versus non-Sidama)

^g^Reference category: Smoking never allowed in home (versus ‘Allowed’ and ‘Not allowed, but exceptions’)

^h^Reference category: No

## Discussion

In this study, we found that overall tobacco use, including both cigarettes and smokeless tobacco products, was extremely low with no women reporting current use and less than 1 % reporting having ever used cigarettes or smokeless tobacco. In addition, the intention to use tobacco was remarkably low with nearly all participants reporting that they had no intention to try either cigarettes or smokeless tobacco in the next year. This is the first study to explore SHS exposure, at the community level, in the Southern region of Ethiopia. While the majority reported that smoking was never allowed in the home (i.e. high level of home smoking bans), one in seven women also reported potential exposure to SHS in their homes on a daily basis. Sociodemographic factors and household-level behaviors associated with reports of SHS exposure in the home were identified and significant differences were observed by place of residence.

Although the sample was homogenous in many ways, significant demographic differences were found based on place of residence or degree of urbanicity in relation to ethnicity, religious affiliation, number of persons per household, level of crowding, years of education, type of house lived in, household decision-making, and in nearly all of the MPI indicators (Table 1). The percentage of the sample classified as “multidimensionally poor” (13.6 %) is considerably lower than that of the national average (87.3 %) and the subnational SNNP regional level (89.7 %), as was reported in the 2011 EDHS [40]. Information on nutritional status was not included in this analysis, however, while the MPI allows for re-weighting of the indicators when these data are not available, the exclusion of nutritional status may have limited the ability to identify additional sources of deprivation. Yet, when compared to the national data, the study sample had lower levels of deprivation in all of the remaining nine indicators, except for use of solid (biomass) fuel for cooking, with a higher level of solid fuel use in the study sample than in the national sample (99.7 % vs. 87 %) [40]. Notably, less than 21 % of the participants were deprived of access to clean drinking water, as compared to more than 66 % of the national sample. The study sample also had higher levels of education, with only 11.3 % deprived in this indicator, as compared to more than 45 % in the national sample [40]. Therefore, based on these indicators, the households in this study sample appear to have a higher standard of living than the national sample. However, when interpreting these findings it should be noted that Lakew & Haile, using national data from the 2011 EDHS, found that adults in the poorest wealth quintile were more likely to use tobacco than those in the richest quintile [41].

### Tobacco use

The tobacco use prevalence rate among women found in this study was lower than the available national data, which reported less than 2 % use of tobacco of any kind [12], and was lower than other regional cross-sectional studies; 0.7 % in Gilgel Gibe, southwest Ethiopia [23], and 0.2 % in eastern Ethiopia [15]. When compared to the findings from the study conducted by Bloch et al., among pregnant women of reproductive age in nine LMICs, from which the current survey was adapted, the ever-tried rate in this sample was also less than that found in the two sub-Saharan African countries included in the original study, namely the Democratic Republic of Congo (14.1 %) and Zambia (6.6 %) [28]. However, it is noted that participants in these two sites in the Bloch et al. study were primarily drawn from large urban cities.

Barriers to the social acceptability of smoking among women are still intact in sub-Saharan African region, and therefore, as has been reported in other LMICs, low social acceptability for female tobacco use and tobacco-related stigma may have led to underreporting of personal tobacco use [1, 28, 42]. However, the overall low smoking rates and low prevalence of smoking among women of reproductive age in this area of Ethiopia highlight the opportunity to focus on primary prevention of tobacco-related diseases among women and their unborn children. Understanding the current social norms associated with smoking, the perceived benefits of all forms of tobacco use, and identification of contextual factors influencing tobacco use, while the prevalence is low, will help to inform the development of tailored primary prevention interventions that take into consideration the unique gender-specific motivations associated with tobacco use, or decision not to use. Given the size and regional variability in Ethioipa and the documented expansion of the tobacco industry, these varied findings underscore the urgent need for comprehensive tobacco use monitoring at the national level as recommended by the WHO FCTC [43].

### *Khat* use

*Khat* use was explored in this study as a covariate, and the ever use prevalence rate among women was the same as the overall prevalence reported nationally in the 2011 EDHS (11 %); however, only 3.4 % of the respondents in this current study who had ever used, reported having chewed *khat* in the last 30 days versus 43 % of among the national sample (Table 2) [12]. There were significant differences between rural versus urban participants, with more urban participants having ever used *khat* as compared to rural participants (*p* < .001). Additionally, *khat* use was associated with reports of daily occurrence of smoking/SHS in the home. These findings support the findings of previous studies that underscore the importance of continuing to monitor the role that *khat* use in this setting plays in relation to tobacco use and subsequently SHS exposure [17–19].

### Secondhand smoke exposure

Less than 8 % of women reported having a member of her household who was currently using tobacco products; however, over 14 % of the total sample, and 22 % of the urban participants, reported that smoking occurred in the home daily. While this item was not a measure of direct exposure to SHS, it does provide an estimate of the potential for daily exposure to SHS in the home. At the same time, only 5 % reported that their young children were “sometimes/frequently” or “always” in close proximity to someone who was smoking in the home. On the surface, there appears to be possible discrepancies between reported number of smokers in the home and reported frequency in which smoking/SHS occurs in the home, with approximately twice as many reports of smoking occurring daily in the home than number of households with a current tobacco user. These findings warrant further exploration, however, possible explanations include guests (non-family members) smoking while visiting, differences in definitions of “family member” and/or intentional efforts to limit young children’s exposure. Additionally, these descrepancies may speak to the presence of tobacco-related stigma and hence a hesitancy on the part of participants to report on family members’ smoking behaviors.

Less than 15 % of the sample reported that smoking was allowed in the their home. This is much less than reported by Reda et al. in the study conducted among a rural population in Eastern Ethiopia, where more than 52 % reported allowing smoking indoors [15]. It is unknown whether the reported low rate of SHS exposure among children (5.1 %), and the high percentage of households in which smoking/SHS is “never allowed” (85.5 %) found in this current study are the result of the overall low smoking prevalence, differences in cultural norms, and/or intentional efforts to communicate and enforce smoking rules and maintain a smoke-free environment in the home. However, it is also possible that the prohibition of smoking in the home represents a dominant cultural norm that could be strengthened through public awareness interventions. Further study would be needed to identify the influencing factors.

In this sample, the strongest predictor of smoking/SHS occurring daily in the home was having a member of the household who used tobacco products. While this may seem intuitive, in a setting where the research on smoking behaviors is nascent, it would be important to be able to document that smokers are smoking indoors in their homes versus in courtyards or outside. Absence of a smoking ban in the home (i.e. “allowing” smoking), and exposure to point-of-sale advertising within the last 30 days, were also predictive of daily occurrence of SHS in the home. Both of these factors have significant policy and programmatic implications, as adoption of indoor smoking bans and restriction of tobacco advertising are among the key evidence-based policy recommendations that have been demonstrated to be the most successful tobacco control policies in the reduction of risk exposure and smoking prevalence across a range of settings, and are required by the WHO FCTC [36, 43].

Interestingly, in this study, while the HDM scores were higher among urban participants and those with higher education [data not presented], the measure did not perform as expected in relation to the whether or not smoking was permitted in the house. The mean composite HDM score for those who reported never allowing smoking in the home was actually lower than the mean HDM score of those that did allow smoking in the home (Table 3). These findings appears to differ from qualitative studies that have described women’s engagement in household decision-making as a factor contributing to adoption of smoke-free homes [44–46]. However, it is noted that the related item only asked whether smoking was “allowed” in the home, and not about the participants’ involvement in establishing or enforcing home smoking rules. There is also evidence in the literature that suggests that the concept of “avoidance self-efficacy” may be more predictive of behaviors associated with SHS exposure [47]. While these findings may also speak to the need for more education in regards to the importance of maintaining a smoke-free home environment, further research is needed to understand the role that awareness, decision-making, self-efficacy, empowerment, and social status play in a woman’s ability to limit her own exposure to SHS and that of her children, particularly in in low-income settings.

### Urbanicity

The stratified analysis of participants by place of residence resulted in a range of observed differences in both outcome variables and covariates. In previous studies increased urbanicity, or differing degrees of interaction and identification with urban settings, has been found to be predictive of more favorable attitudes toward smoking [27, 42, 48]. Urban settings have also been associated with an increased prevalence of smoking among women both in high and low-income countries [49]. If the EDHS criteria is applied, the entire sample in this study would be considered rural; yet, even this relatively small degree of difference in place of residence (i.e., residents from a small rural town compared to those from the immediate outlying rural districts) resulted in an increased likelihood of smoking/SHS occurring daily in the home, even after controlling for other factors (Table 3) [12].

A number of reasons for the observed urban-rural differentials have been considered. Overall, the urban participants had less sources of deprivation, and higher levels of education than the rural residents, which may provide greater levels of expendable income that can be used for tobacco products. This observed difference may also be attributed to differences in social network tobacco-use norms in more urbanized settings. In a study conducted in 2008, among women in South Africa, Williams et al. found urbanicity to have an independent effect on smoking-related attitudes [42]. In addition, urbanicity moderated the effect of network smoking norms on smoking related attitudes; but, it did not moderate cigarette advertising exposure. In a more recent study, also conducted in South Africa, smoking prevalence among women was associated with having spent more than half of their lives in urban settings (*p* < .001), coping poorly with stress, and an increase in adverse life events; however, these factors were not significant among men [50]. On the other hand, being poor was significantly associated with a higher smoking prevalence among both men (*p* = .024) and women (*p* = .002), while education level, employment status, and housing quality were not found to be significantly associated with smoking prevalence for men or women [50].

While no studies conducted in sub-Saharan Africa were found which reported rural versus urban differences in SHS exposure, a number of studies from India have reported significantly higher levels of SHS exposure, in both household and workplace environments, among households living in rural versus urban settings [51, 52]. Predictors of smoking have also varied based on place of residence; Singh and Sahoo found place of residence to be the strongest predictor among participants living in rural areas, while education was the most significant among participants in urban settings [51]. These divergent findings indicate that further exploration is needed to elucidate factors in both rural and urban settings that are contributing to differences in smoking prevalence and indoor smoking-related behaviors.

In this study, significant differences were noted between rural and urban reports of involvement in household decision-making, with the urban participants reporting more involvement in decision-making in all areas (*p* < .001). However, it is noted that the reported level of urban women’s involvement in decisions related to major household purchases (54 %) and visitation of family and friends (71.3 %) are still lower than that reported by women at the national level (66.2 and 78 % respectively) [12]. The 2011 EDHS used a 4-item version of the household-decision making scale, which included an additional item on decisions related to accessing healthcare. Use of a different version of the tool may account for this difference; however, comparisons have yet to be made between these two versions of the tool. Another explanation for this difference may be the degree of urbanicity; if household decision-making increases with urbanicity, the lower level of household decision-making among this sample may be related to overall lower levels of urbanicity. However, these findings underscore the need for further research to understand the relationship between urbanicity, empowerment, decision-making, and SHS exposure.

Participants from the urban *kebeles* differed significantly from rural participants in relation to ethnicity, with greater representation from various ethnic groups, level of adoption of smoke-free homes (i.e. smoking “never allowed”), exposure to point-of-sale advertising, and prevalence of ever *khat* use. In bivariate analysis, each of these factors were also significantly associated with daily occurrence of smoking/SHS in the home. These factors were not highly correlated (*r* < .50) with each other and thereby may begin to help characterize factors in the more urbanized environments that are influencing tobacco use and risk of SHS exposure. For example, the greater percentage of non-Sidama ethnicities found within the urban *kebeles* may be indicative of migration from other areas of the country that have different social norms concerning tobacco use and SHS exposure [42].

A number of recent studies from China and India have demonstrated a strong association between rural-to-urban migration with an increase in smoking prevalence [53–56]. Migration has been associated with changes in social networks and influences, changes in self-definition, as well as increased vulnerability to a range of other health risks which may also begin to give insight into the observed differences in rates of smoking and indoor SHS exposure [42, 57]. An emerging body of knowledge outlines the unique risk factors that can be attributed to urban settings [57–60]. These risk factors can vary based on differences in social, cultural and physical environmental contexts [57]. Additionally, factors such as gender and ethnicity can mediate these risk factors [58]. Therefore, it is critical that tobacco control policies and interventions be informed by local data. Furthermore, the findings from this study provide support for prioritization of tobacco use prevention efforts in both rural towns and *emerging* urban areas. Further research is needed to understand the dynamics that contribute to the increased rates of smoking by household members and SHS exposure in these settings. Additionally, there is a need to standardize methods used to characterize neighborhoods and communities, and to incorporate more complex evaluation of urban-rural differences versus simple binary urban-rural measures, in order to generate comparable data across studies [60, 61].

### Strengths and limitations

This study adds to an emerging body of evidence on women’s tobacco use and SHS exposure in Ethiopia. The study was strengthened by the use of items from standardized validated instruments, and use of translation and adaptation guidelines that have been used previously in LMICs, [28, 32, 35, 62]. The generalizability was enhanced by use of a systematic sampling technique to select households and the reliability of the self-report of SHS exposure was strengthened by use of an interviewer-administered survey [63].

At the same time, several limitations of this study should be considered. First, the outcome variables relied on self-report, which can be susceptible to a number of non-sampling errors. However, self-report methodology has been used extensively in tobacco-related research and has been demonstrated to yield accurate estimates of prevalence in validation studies using biomarkers [36, 63]. Furthermore, in this study a number of recommended steps were taken to mitigate these types of error, including careful consideration of question wording assurance of privacy and confidentiality, and use of an interviewer-administered questionnaire to improve accuracy [36, 63, 64]. An interviewer-administered survey also increases the inclusion of women with low literacy levels, yet, it is noted that this may also have introduced social acceptability bias, particularly in settings, such as this one, with a potential for high levels of tobacco-related stigma and defined gender roles that limit women’s autonomy, thereby potentially resulting in underreporting of personal and/or family member tobacco use. Second, during the data collection exercises, approximately 20 % of the households approached (*n* = 137) were not at home. This introduces a potential for selection bias, as it is unknown if these residents may have differed in some way from participants that were home during the day. Additionally, while differences were found between rural and urban *kebeles*, rural *kebeles* still had relatively close proximity to the town (i.e., less than one hour walking distance) and it is unknown whether the other *kebeles* in the Aleta Wondo district, which are further away from town, may have differed in the variables measured. Finally, the small sample size prevented the testing of 2-way interactions in the logistic regression model.

## Conclusions

The findings from this study represent an important contribution to the current understanding of SHS exposure among rural women and children in Southern Ethiopia. While the reported prevalence of daily SHS in the home was lower (14.4 %) than found in the previous study conducted in Eastern Ethiopia (52 %) [15], the current rate of exposure continues to represent an increased risk profile for women and children, particularly in a population already at high risk of exposure to household air pollution from use of solid fuels for cooking and heating [40]. Additionally, while the differences observed between the reports of tobacco users in the home and the frequency of smoking in the home may be a result of stigma associated with tobacco use resulting in underreporting by family members, these findings continue to provide a useful starting point for understanding tobacco-related behavior among women in this region of Ethiopia. They also suggest the need for replication in other areas.

The findings from this study can help to inform the development of contextualized gender-specific primary prevention tobacco control interventions, particularly in relation to the promotion of smoke-free homes. Living with a tobacco user, allowing smoking in the home, living in a more urbanized setting, and exposure to point-of-sale advertising were all found to be associated with daily smoking/SHS in the home. Based on research in other LMICs, in areas where enhanced levels of awareness of health risks were associated with tobacco use and SHS exposure, and increased exposure to anti-tobacco messaging exist, basic awareness measures (including surveillance activities) could lead to significant increases in adoption of household smoking bans, especially among households with no smokers [65]. In turn, higher adoption of smoking bans would also have the potential to proactively influence dominant community norms related to tobacco use and SHS exposure.

This study represents a response to the wide call from public health experts to seize the “window of opportunity” to proactively address the looming tobacco epidemic and increase in NCD-associated burden of disease being projected for the sub-Saharan African region, particularly for women [28, 66, 67]. Public health interventions are urgently needed in Ethiopia, in both urban and rural environments, in order to counter the influence of the growing tobacco industry in Ethiopia. However, in order to develop tailored interventions for these settings further research is needed to identify and understand the role that contextual factors in this environment may have on the prevention of tobacco use and SHS exposure among women and children.
